# Supplementary material for: Learning neural network potentials from experimental data via Differentiable Trajectory Reweighting
Source: Nat Commun. 2021 Nov 25;12:6884. doi: 10.1038/s41467-021-27241-4 (PMC8617111; doi:10.1038/s41467-021-27241-4)
Supplement: Supplementary file 1 — Supplementary Information [file 41467_2021_27241_MOESM1_ESM.pdf]

Learning neural network potentials from experimental data via Differentiable Trajectory Reweighting: Supplementary Information

Stephan Thaler[1] and Julija Zavadlav[1,2]

[1]*Professorship of Multiscale Modeling of Fluid Materials, TUM School of Engineering and Design, Technical University of Munich, Germany*
[2]*Munich Data Science Institute, Technical University of Munich, Germany*

Contents

Supplementary Methods **1**
1. DiffTRe and simulation parameters 1
   1.1. Double-well toy example 1
   1.2. Atomistic model of diamond 2
   1.3. Coarse-grained water model 2
2. Speed-up considerations 2
3. Continuously differentiable binning 2
4. Stress-strain relations 3
5. Derivation of the gradient 4
6. DimeNet++ hyperparameters 5

Supplementary Figures **5**

Supplementary References **13**

Supplementary Methods

1. DiffTRe and simulation parameters

First, we summarize DiffTRe parameters relevant to all examples before we list problem-specific parameters below. We have set $\bar{N}_{\text{eff}} = 0.9N$ as the threshold above which re-using a trajectory is allowed. We employ an Adam optimizer [1] with exponentially decaying learning rate. Adam hyperparameters $\beta_1 = 0.1$ and $\beta_2 = 0.4$ are chosen to account for training with rather large step sizes and only few parameter updates. All examples are initialized with a global random seed 0, which controls the random initialization of $\boldsymbol{\theta}$ and the initial simulation state. We observed that despite setting random seeds, results are not matched exactly across different re-runs – even when running JAX on reproducibility configuration. We tackle this issue by reporting results for varying random seeds that also capture variability from non-deterministic operations. All computations are run on a single Nvidia RTX 3090 GPU with the exception of computations with the cubic spline potential in the double-well toy example. As the numerically inexpensive spline cannot saturate the GPU, computations were faster on an AMD Ryzen Threadripper 3070X CPU.

1.1. Double-well toy example

Simulations consist of $N_p = 2000$ ideal gas particles of mass $m = 1$ within a box of size $X = 1$ and time step $\delta t = 0.001$. The constant temperature of $k_B T = 1$ in the canonical ensemble is enforced by a Nose-Hoover chain thermostat [2] with 5 chains and time scale $\tau = 0.02$. The initial state $\mathbf{S}_{\text{init}}$ is constructed by randomly drawing particles uniformly from $x \in [0, 1]$. $\mathbf{S}_{\text{init}}$ for the final production run consists of particles drawn uniformly from $x \in [0.5, 0.51]$ to test convergence to the target density distribution, even from a state far from equilibrium. Density distributions are computed via the differentiable density function in Supplementary Eq. (4) with bin width

$\Delta x = 0.01$. During optimization, the initial learning rate $\eta = 0.5$ of Adam [1] is decayed exponentially by a factor of 0.01 over 200 update steps. The target and final predicted densities $\tilde{\rho}(x)/\rho_0$ and $\rho(x)/\rho_0$ are computed based on a production run of 100000 states following 10000 skipped states for equilibration.

1.2. Atomistic model of diamond

Simulations are run with a time step size of $\delta t = 0.5$ fs. The temperature is controlled by a Langevin thermostat with friction coefficient $\gamma = 4/$ ps, which corresponds to a coupling time scale of $250fs$. These values are common in simulations of diamond in the literature [3]. Carbon atoms have a mass $m = 12.011$ u. The loss weights $\gamma_{\boldsymbol{\sigma}} = 5 \cdot 10^{-8}(\frac{\mathrm{kJ}}{\mathrm{mol\ nm}^3})^{-2}$ and $\gamma_{\mathbf{C}} = 10^{-10}(\frac{\mathrm{kJ}}{\mathrm{mol\ nm}^3})^{-2}$ balance the impact of both observables, i.e. stress $\boldsymbol{\sigma}$ and stiffness values C_{ij}. Optimization starts with an initial Adam learning rate $\eta = 0.002$ that is exponentially decayed by a factor of 0.01 over 500 steps.

In computation of phonon density of states (PDOS), we minimize the potential energy via 500 steps of the Fast Inertial Relaxation Engine (FIRE) [4]. PDOS is computed afterwards via the finite displacement method as implemented in Phonopy [5] with displacement length 0.001 nm.

1.3. Coarse-grained water model

Coarse-grained water is simulated with a time step size of $\delta t = 2$ fs. Water molecules (and CG water particles correspondingly) have a mass $m = 18.0154$ u. A Nose-Hoover chain thermostat [2] with chain length 5 and time scale $\tau = 200$ fs enforces the target temperature. We approximate radial (RDF) and angular distribution functions (ADF) with the differentiable versions presented in Supplementary Eq. (5) and (6). The RDF is discretized by 300 bins of width $\Delta x = \frac{1}{300}$ nm. The ADF is discretized by 200 bins of width $\Delta \alpha = \frac{\pi}{200}$ rad and triplets are cut off at $r_c = 0.318$ nm analogous to the experimental evaluation [6]. The loss weight $\gamma_p = 10^{-7}(\frac{\mathrm{kJ}}{\mathrm{mol\ nm}^3})^{-2}$ accounts for the larger magnitude of pressure versus the RDF and ADF. The initial Adam learning rate $\eta = 0.003$ is decayed exponentially by a factor of 0.01 over 200 steps.

The tetrahedral order parameter q [7] is computed via the triplet angles α_{ijk} spanned by neighboring particles i and k of a central particle j. i and k are indices running over the 4 nearest neighbors of particle i and

$$q = 1 - \frac{3}{8} \sum_{i=1}^{3} \sum_{k=i+1}^{4} \left(\cos \alpha_{ijk} + \frac{1}{3} \right)^2 . \tag{1}$$

We compute the self-diffusion coefficient D via the Green-Kubo relation from the velocity auto-correlation function (VACF)

$$D = \frac{1}{3} \int_0^{t_{\mathrm{cut}}} \left\langle \frac{1}{N_p} \sum_{i=1}^{N_p} \mathbf{v}_i(t_0) \cdot \mathbf{v}_i(t_0 + \tau) \right\rangle_{t_0} \mathrm{d}\tau \ , \tag{2}$$

where we cut the VACF at $t_{\mathrm{cut}} = 1$ ps to reduce the effect of spurious long-term non-zero correlations. N_p is the number of particles in the box.

2. Speed-up considerations

Assuming a numerically expensive (NN) potential dominating computational effort, s_g is determined by the cost of necessary force evaluations during trajectory generation per retained state energy computation: As forces for NN potentials are computed by backpropagating potential energy values, they are approximately G times as expensive as energy computations. The provided rule-of-thumb formula in the main text overestimates s_g for expensive observables, but systematically underestimating s_g by ignoring the cost of backpropagating through time integrator operations. Recognizing that gradient computation costs with DiffTRe are negligible compared to reference trajectory generation costs (under the same assumption of numerically cheap observables), $s \sim G + 1$ reflects the cost of trajectory generation plus backward pass versus only the trajectory generation in the case of DiffTRe. We presumed a value of $G \approx 3$ for the given estimates in the toy example, which mirrors that gradient computation in the adjoint method requires integrating 3 ordinary differential equations backwards in time [8].

3. Continuously differentiable binning

The (discrete) Dirac function used in binning can be substituted by a Gaussian probability density function (PDF) centered at position x_k of binned entity k. The value of bin $b_k(x)$ centered at x can be computed as

$$b_k(x) = \Delta x * s_k(x) \quad \text{with} \quad s_k(x) = \frac{1}{\sqrt{2\pi\delta^2}} e^{-\frac{(x-x_k)^2}{2\delta^2}} \ , \tag{3}$$

where Δx is the bin width. The implied discrete integral over a PDF guarantees an overall contribution of unity for each binned entity. We set the Gaussian standard deviation $\delta = \Delta x$. For a fine grid $\delta \to 0$, the Dirac function is recovered.

Eq. (3) allows defining a normalized differentiable density function

$$\rho(x) \simeq \frac{1}{N_p} \sum_{k=1}^{N_p} b_k(x) \ , \tag{4}$$

where x_k is the position of each particle in the simulation and N_p is the number of particles in the box. Analogously, we can define

$$RDF(d) \simeq \frac{\Omega}{V(d) N_p^2} \sum_{k=1}^{N_{\text{pair}}} b_k(d) \ , \tag{5}$$

where $V(d)$ is the volume of the sphere shell of $b_k(d)$ and Ω is the simulation box volume.

The ADF is a probability density function (PDF) over triplet angles α_{ijk} for all particle triplets ijk within a cut-off radius r_c of central particle j. We smooth the radial cut-off via a Gaussian cumulative distribution function (CDF) $\Phi(r; r_c, \sigma^2)$ centered at r_c with variance σ^2.

$$ADF(\alpha) \simeq \frac{\overline{ADF}(\alpha)}{\int_0^\pi \overline{ADF}(\alpha) \mathrm{d}\alpha} \quad \text{with} \quad \overline{ADF}(\alpha) = \sum_{k=1}^{N_{\text{triplet}}} (1 - \Phi(r_{k,\max}; r_c, \sigma^2)) b_k(\alpha) \ , \tag{6}$$

where $r_{k,\max} = \max(r_{ij}, r_{kj})$.

4. Stress-strain relations

Voigt notation provides a convenient way to describe the stress-strain relation by reducing pairs of indices to single digits: $11 \mapsto 1$, $22 \mapsto 2$, $33 \mapsto 3$, $23 \mapsto 4$, $13 \mapsto 5$, and $12 \mapsto 6$. Generalized Hooke's law can then be written as

$$\sigma_i = \mathbf{C}_{ij} \epsilon_j \quad \text{with} \quad \boldsymbol{\sigma} = \begin{pmatrix} \sigma_1 \\ \sigma_2 \\ \sigma_3 \\ \sigma_4 \\ \sigma_5 \\ \sigma_6 \end{pmatrix} = \begin{pmatrix} \sigma_{11} \\ \sigma_{22} \\ \sigma_{33} \\ \sigma_{23} \\ \sigma_{13} \\ \sigma_{12} \end{pmatrix} \ ; \quad \boldsymbol{\epsilon} = \begin{pmatrix} \epsilon_1 \\ \epsilon_2 \\ \epsilon_3 \\ \epsilon_4 \\ \epsilon_5 \\ \epsilon_6 \end{pmatrix} = \begin{pmatrix} \epsilon_{11} \\ \epsilon_{22} \\ \epsilon_{33} \\ 2\epsilon_{23} \\ 2\epsilon_{13} \\ 2\epsilon_{12} \end{pmatrix} , \tag{7}$$

assuming $\boldsymbol{\sigma} = \mathbf{0}$ for $\boldsymbol{\epsilon} = \mathbf{0}$. Due to the symmetry in the diamond cubic crystal system, Eq. (7) simplifies to only 3 distinct values in $\mathbf{C}$

$$\begin{pmatrix} \sigma_1 \\ \sigma_2 \\ \sigma_3 \\ \sigma_4 \\ \sigma_5 \\ \sigma_6 \end{pmatrix} = \begin{pmatrix} C_{11} & C_{12} & C_{12} & 0 & 0 & 0 \\ & C_{11} & C_{12} & 0 & 0 & 0 \\ & & C_{11} & 0 & 0 & 0 \\ & & & C_{44} & 0 & 0 \\ & \text{sym} & & & C_{44} & 0 \\ & & & & & C_{44} \end{pmatrix} \begin{pmatrix} \epsilon_1 \\ \epsilon_2 \\ \epsilon_3 \\ \epsilon_4 \\ \epsilon_5 \\ \epsilon_6 \end{pmatrix} . \tag{8}$$

The inverse relation is defined by the compliance tensor $\mathbf{S} = \mathbf{C}^{-1}$, which is usually given in terms of Young's modulus E, shear modulus G and Poisson's ratio ν

$$\begin{pmatrix} \epsilon_1 \\ \epsilon_2 \\ \epsilon_3 \\ \epsilon_4 \\ \epsilon_5 \\ \epsilon_6 \end{pmatrix} = \begin{pmatrix} \frac{1}{E} & \frac{-\nu}{E} & \frac{-\nu}{E} & 0 & 0 & 0 \\ & \frac{1}{E} & \frac{-\nu}{E} & 0 & 0 & 0 \\ & & \frac{1}{E} & 0 & 0 & 0 \\ & & & \frac{1}{G} & 0 & 0 \\ & \text{sym} & & & \frac{1}{G} & 0 \\ & & & & & \frac{1}{G} \end{pmatrix} \begin{pmatrix} \sigma_1 \\ \sigma_2 \\ \sigma_3 \\ \sigma_4 \\ \sigma_5 \\ \sigma_6 \end{pmatrix} . \tag{9}$$

The stress-strain curves are computed by deforming the box in two separate modes that yield states of pure normal and shear strain, respectively [9]. In the normal mode, we transform the box according to

$$\begin{pmatrix} X \\ Y \\ Z \end{pmatrix} \rightarrow \begin{pmatrix} X(1+\xi) \\ Y \\ Z \end{pmatrix} , \tag{10}$$

which yields the non-zero strain $\epsilon_1 = \epsilon_{11} = \xi$ in the strain vector $\boldsymbol{\epsilon} = (\epsilon_1, 0, 0, 0, 0, 0)$. A pure shear mode is given by the transformation

$$\begin{pmatrix} X \\ Y \\ Z \end{pmatrix} \rightarrow \begin{pmatrix} X \\ Y + Z\xi \\ Z \end{pmatrix} , \tag{11}$$

which yields $\epsilon_4 = 2\epsilon_{23} = \xi$ in the strain vector $\boldsymbol{\epsilon} = (0, 0, 0, \epsilon_4, 0, 0)$. These elementary deformations [9] allow probing $\mathbf{C}$ such that a single component of $\mathbf{C}$ describes the relation between ϵ_i and measured σ_j (Eq. (8))

$$\sigma_1 = C_{11}\epsilon_1 \ ; \quad \sigma_2 = C_{12}\epsilon_1 \ ; \quad \sigma_4 = C_{44}\epsilon_4 \ . \tag{12}$$

5. Derivation of the gradient

$$L(\boldsymbol{\theta}) = (\langle O(U_{\boldsymbol{\theta}})\rangle - \tilde{O})^2 \simeq \left(\sum_{i=1}^{N} w_i O(\mathbf{S}_i, U_{\boldsymbol{\theta}}) - \tilde{O} \right)^2 = \bar{L}(\boldsymbol{\theta}) \tag{13}$$

$$\frac{\partial \bar{L}}{\partial \boldsymbol{\theta}} = 2 \Bigg(\underbrace{\sum_{i=1}^{N} w_i O(\mathbf{S}_i, U_{\boldsymbol{\theta}})}_{\simeq \langle O(U_{\boldsymbol{\theta}})\rangle} - \tilde{O} \Bigg) \frac{\partial}{\partial \boldsymbol{\theta}} \sum_{i=1}^{N} w_i O(\mathbf{S}_i, U_{\boldsymbol{\theta}}) \tag{14}$$

$$\sum_{i=1}^{N} \frac{\partial}{\partial \boldsymbol{\theta}} (w_i O(\mathbf{S}_i, U_{\boldsymbol{\theta}})) = \sum_{i=1}^{N} \frac{\partial w_i}{\partial \boldsymbol{\theta}} O(\mathbf{S}_i, U_{\boldsymbol{\theta}}) + \underbrace{\sum_{i=1}^{N} w_i \frac{\partial O(\mathbf{S}_i, U_{\boldsymbol{\theta}})}{\partial \boldsymbol{\theta}}}_{\simeq \langle \frac{\partial O(U_{\boldsymbol{\theta}})}{\partial \boldsymbol{\theta}} \rangle} \tag{15}$$

$$\begin{aligned}
\frac{\partial w_i}{\partial \boldsymbol{\theta}} &= \frac{\partial}{\partial \boldsymbol{\theta}} \left(\frac{e^{-\beta(U_{\boldsymbol{\theta}}(\mathbf{S}_i) - U_{\hat{\boldsymbol{\theta}}}(\mathbf{S}_i))}}{\sum_{j=1}^{N} e^{-\beta(U_{\boldsymbol{\theta}}(\mathbf{S}_j) - U_{\hat{\boldsymbol{\theta}}}(\mathbf{S}_j))}} \right) \\
&= \underbrace{\frac{e^{-\beta(U_{\boldsymbol{\theta}}(\mathbf{S}_i) - U_{\hat{\boldsymbol{\theta}}}(\mathbf{S}_i))}}{\sum_{j=1}^{N} e^{-\beta(U_{\boldsymbol{\theta}}(\mathbf{S}_j) - U_{\hat{\boldsymbol{\theta}}}(\mathbf{S}_j))}}}_{w_i} \left(-\beta \frac{\partial U_{\boldsymbol{\theta}}(\mathbf{S}_i)}{\partial \boldsymbol{\theta}} \right) + \frac{e^{-\beta(U_{\boldsymbol{\theta}}(\mathbf{S}_i) - U_{\hat{\boldsymbol{\theta}}}(\mathbf{S}_i))}}{-\left(\sum_{j=1}^{N} e^{-\beta(U_{\boldsymbol{\theta}}(\mathbf{S}_j) - U_{\hat{\boldsymbol{\theta}}}(\mathbf{S}_j))} \right)^2} \\
&\quad * \sum_{j=1}^{N} \left[e^{-\beta(U_{\boldsymbol{\theta}}(\mathbf{S}_j) - U_{\hat{\boldsymbol{\theta}}}(\mathbf{S}_j))} \left(-\beta \frac{\partial U_{\boldsymbol{\theta}}(\mathbf{S}_j)}{\partial \boldsymbol{\theta}} \right) \frac{\sum_{k=1}^{N} e^{-\beta(U_{\boldsymbol{\theta}}(\mathbf{S}_k) - U_{\hat{\boldsymbol{\theta}}}(\mathbf{S}_k))}}{\sum_{k=1}^{N} e^{-\beta(U_{\boldsymbol{\theta}}(\mathbf{S}_k) - U_{\hat{\boldsymbol{\theta}}}(\mathbf{S}_k))}} \right] \\
&= w_i \left(-\beta \frac{\partial U_{\boldsymbol{\theta}}(\mathbf{S}_i)}{\partial \boldsymbol{\theta}} \right) + \frac{e^{-\beta(U_{\boldsymbol{\theta}}(\mathbf{S}_i) - U_{\hat{\boldsymbol{\theta}}}(\mathbf{S}_i))}}{-\left(\sum_{j=1}^{N} e^{-\beta(U_{\boldsymbol{\theta}}(\mathbf{S}_j) - U_{\hat{\boldsymbol{\theta}}}(\mathbf{S}_j))} \right)^2} \\
&\quad * \sum_{k=1}^{N} e^{-\beta(U_{\boldsymbol{\theta}}(\mathbf{S}_k) - U_{\hat{\boldsymbol{\theta}}}(\mathbf{S}_k))} \sum_{j=1}^{N} \Bigg[\left(-\beta \frac{\partial U_{\boldsymbol{\theta}}(\mathbf{S}_j)}{\partial \boldsymbol{\theta}} \right) \underbrace{\frac{e^{-\beta(U_{\boldsymbol{\theta}}(\mathbf{S}_j) - U_{\hat{\boldsymbol{\theta}}}(\mathbf{S}_j))}}{\sum_{k=1}^{N} e^{-\beta(U_{\boldsymbol{\theta}}(\mathbf{S}_k) - U_{\hat{\boldsymbol{\theta}}}(\mathbf{S}_k))}}}_{w_j} \Bigg] \\
&= w_i \left(-\beta \frac{\partial U_{\boldsymbol{\theta}}(\mathbf{S}_i)}{\partial \boldsymbol{\theta}} \right) + \underbrace{\frac{e^{-\beta(U_{\boldsymbol{\theta}}(\mathbf{S}_i) - U_{\hat{\boldsymbol{\theta}}}(\mathbf{S}_i))}}{-\sum_{j=1}^{N} e^{-\beta(U_{\boldsymbol{\theta}}(\mathbf{S}_j) - U_{\hat{\boldsymbol{\theta}}}(\mathbf{S}_j))}}}_{-w_i} * \sum_{j=1}^{N} w_j \left(-\beta \frac{\partial U_{\boldsymbol{\theta}}(\mathbf{S}_j)}{\partial \boldsymbol{\theta}} \right)
\end{aligned} \tag{16}$$

$$\sum_{i=1}^{N} \frac{\partial w_i}{\partial \boldsymbol{\theta}} O(\mathbf{S}_i, \boldsymbol{\theta}) = \sum_{i=1}^{N} w_i \left(-\beta \frac{\partial U_{\boldsymbol{\theta}}(\mathbf{S}_i)}{\partial \boldsymbol{\theta}} \right) O(\mathbf{S}_i, \boldsymbol{\theta}) + \sum_{i=1}^{N} -w_i O(\mathbf{S}_i, \boldsymbol{\theta}) \sum_{j=1}^{N} w_j \left(-\beta \frac{\partial U_{\boldsymbol{\theta}}(\mathbf{S}_j)}{\partial \boldsymbol{\theta}} \right) \tag{17}$$

$$\simeq \langle -\beta \frac{\partial U_{\boldsymbol{\theta}}}{\partial \boldsymbol{\theta}} O(U_{\boldsymbol{\theta}}) \rangle + \langle -O(U_{\boldsymbol{\theta}}) \rangle \langle -\beta \frac{\partial U_{\boldsymbol{\theta}}}{\partial \boldsymbol{\theta}} \rangle \tag{18}$$

$$\Rightarrow \frac{\partial \bar{L}}{\partial \boldsymbol{\theta}} \simeq 2(\langle O(U_{\boldsymbol{\theta}}) \rangle - \tilde{O}) \left[\langle \frac{\partial O(U_{\boldsymbol{\theta}})}{\partial \boldsymbol{\theta}} \rangle - \beta \left(\langle O(U_{\boldsymbol{\theta}}) \frac{\partial U_{\boldsymbol{\theta}}}{\partial \boldsymbol{\theta}} \rangle - \langle O(U_{\boldsymbol{\theta}}) \rangle \langle \frac{\partial U_{\boldsymbol{\theta}}}{\partial \boldsymbol{\theta}} \rangle \right) \right] = \frac{\partial L}{\partial \boldsymbol{\theta}} \tag{19}$$

6. DimeNet++ hyperparameters

We refer the reader to the original DimeNet / DimeNet++ publications [10, 11] for a detailed description of the neural network architecture. We reduced embedding sizes by factor 4: The standard embedding size then becomes 32, the output embedding size 64, the triplet and atom-type embedding size becomes 16 and the Bessel-basis embedding remains at a size of 8. All other hyperparameters are unchanged: A cut-off length of 0.5 nm (0.2 nm for diamond), 4 interaction layers, 3 fully-connected output layers, 1 residual block before and 2 residual blocks after the skip connection, 6 radial and 7 angular Bessel embedding function with a continuously differentiable envelope function of order 6 and a swish [12] activation function. Weights are initialized via an orthogonal Glorot[13, 10] scheme.

Supplementary Figures

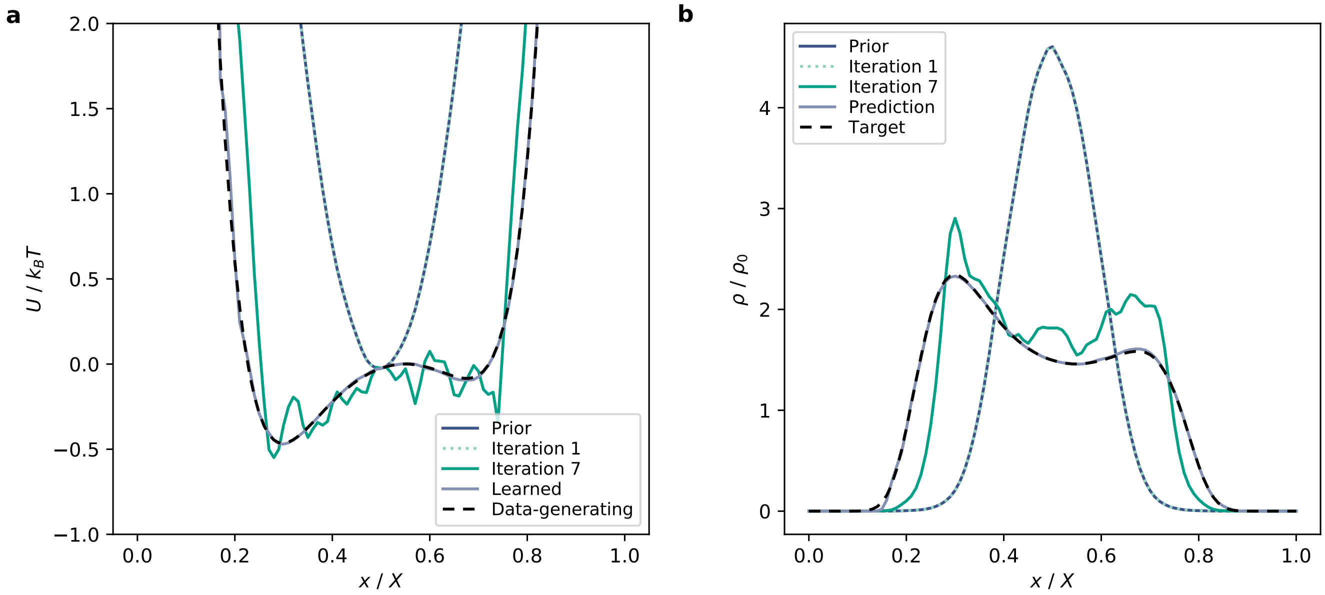

Figure 1: Double-well toy example across optimization. By learning the normalized density, DiffTRe adjusts $U_{\boldsymbol{\theta}}^{\text{model}}$ such that $U^{\text{prior}} + U_{\boldsymbol{\theta}}^{\text{model}}$ eventually recovers the data-generating potential (**a**). Accordingly, the corresponding predicted normalized densities converge to the target (**b**). Potentials in panel **a** are shifted vertically for visualization purposes such that all potentials coincide at $x/X = 0.5$.

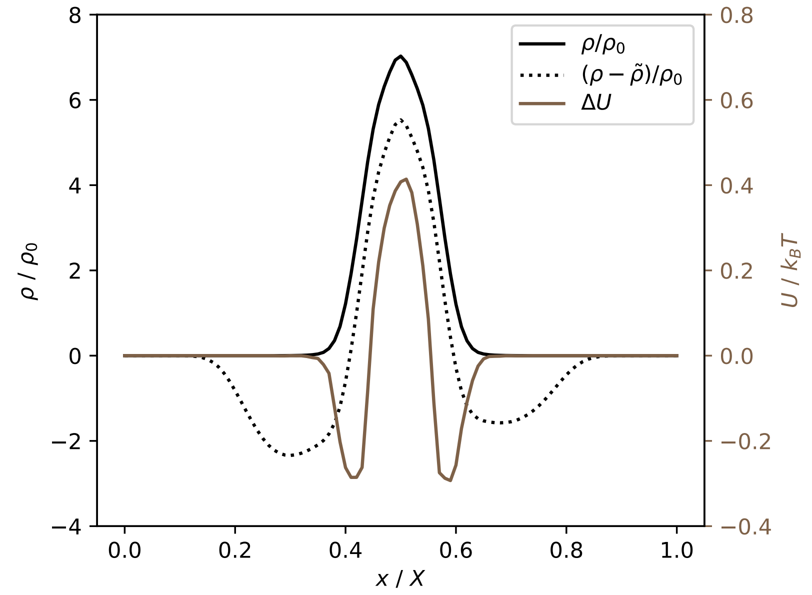

Figure 2: Double-well toy example vanishing gradients. There are areas on the potential energy surface (PES) where the effect of the gradient on the PES $\Delta U = U(\boldsymbol{\theta} - \nabla_{\boldsymbol{\theta}} L) - U(\boldsymbol{\theta}) = 0$, even though these areas contribute to the loss ($\rho - \tilde{\rho} \neq 0$). This is due to the reference trajectory that contains no states in these areas of the PES ($\rho = 0$ and $\nabla_{\boldsymbol{\theta}} \rho = 0$; compare Supplementary Eq. (19)).

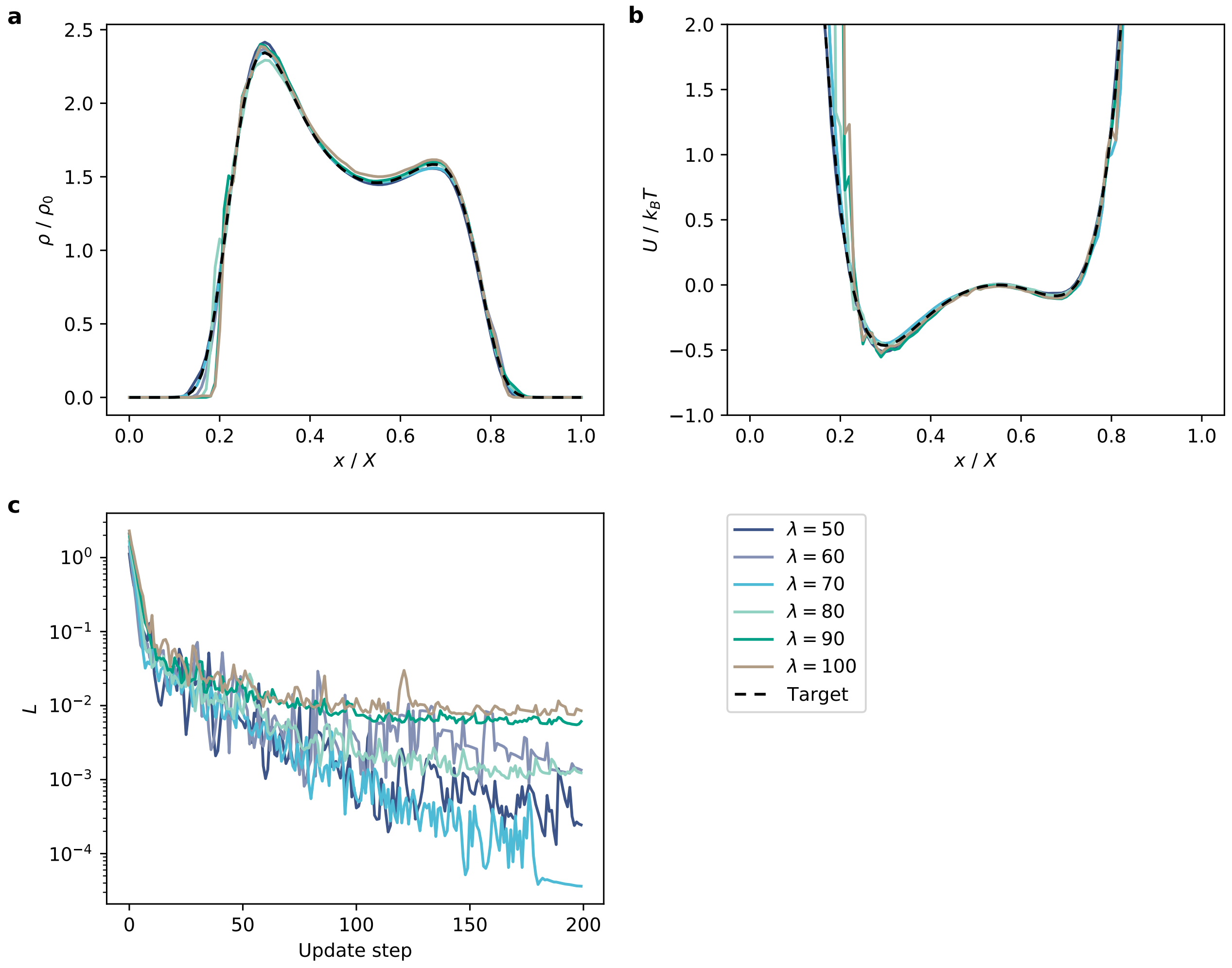

Figure 3: Double-well prior variation study. Resulting density (**a**) and learned potential (**b**) with respective targets for varying prior scales λ. The prior loss curves (**c**) show the impact of the prior on the initial loss value $L(0)$ and optimization convergence. Many possible λ lead to satisfactory learning outcomes ($\mathbf{a}-\mathbf{b}$).

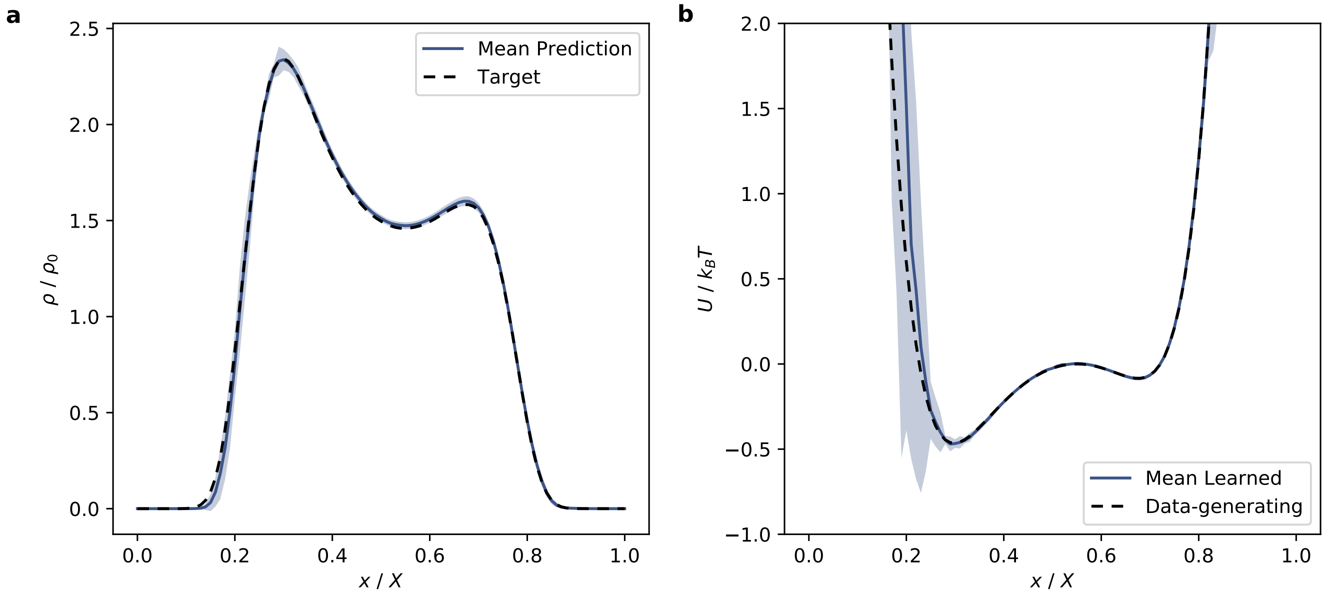

Figure 4: Random initialization study for the double-well toy example. A mean matching the target and small standard deviations (shaded area) when re-starting the optimization with random seeds from $0 - 99$ demonstrates that the learned normalized density profile is robust with respect to initialization of the spline and the initial simulation state (**a**). The corresponding learned potential exhibits larger standard deviations at the left well boundary due to difficult training in this region (**b**). Potentials are shifted vertically for visualization purposes such that all potentials coincide at $x/X = 0.5$.

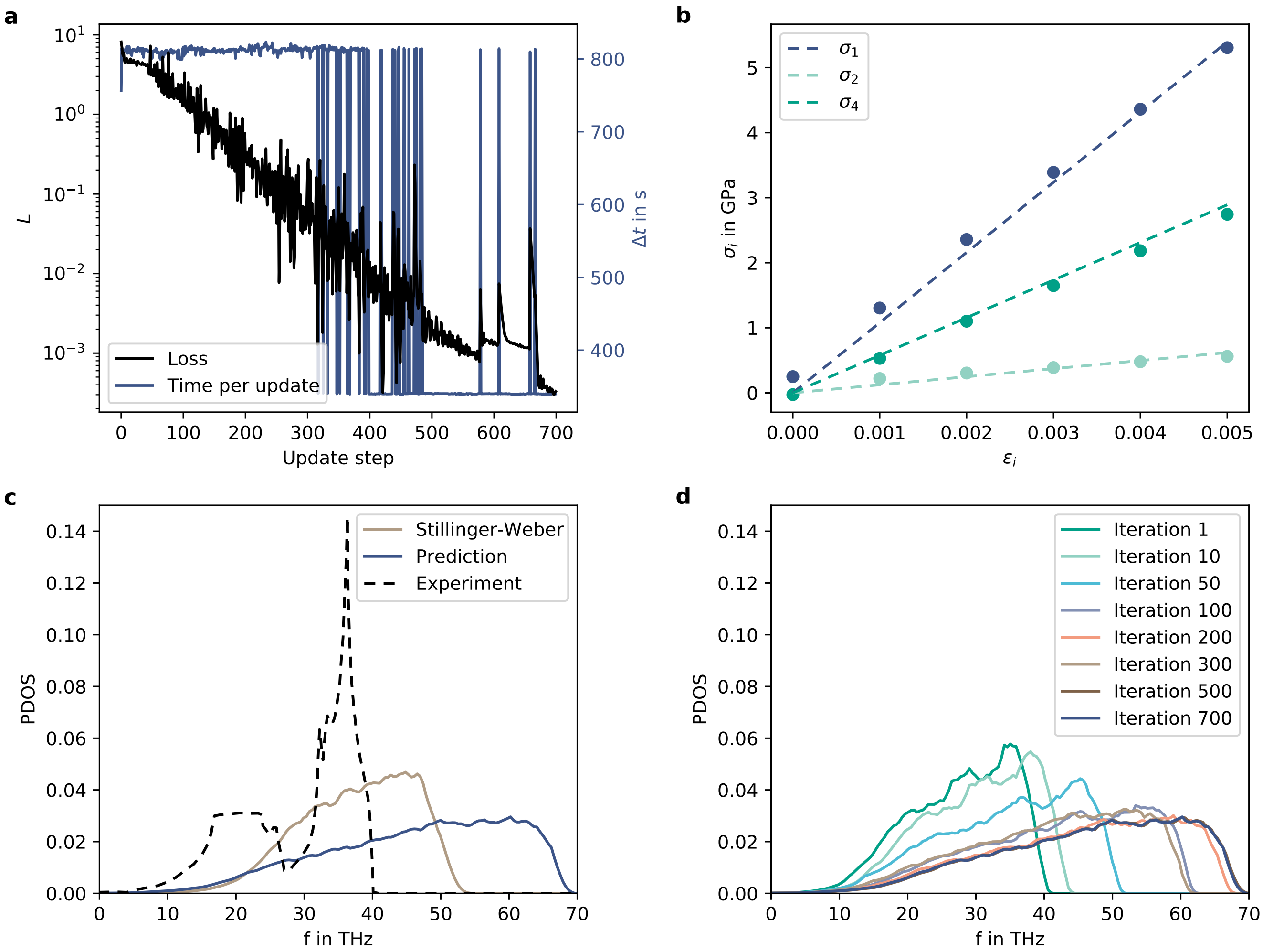

Figure 5: Supplementary results for the diamond model. The large reduction in the loss L confirms successful learning (**a**). Reduction in wall-clock time per parameter update Δt in the second half of the optimization is achieved through re-using previously generated trajectories. Panel **b** displays an alternative stiffness computation method, explicit box deformation. Assuming a linear stress-strain relationship for small $\boldsymbol{\epsilon}$ and a perfect alignment of the learned potential with experimental $\tilde{\boldsymbol{\sigma}} = \mathbf{0}$ and $\tilde{C}_{ij}$, all measured σ_i lie on the respective dashed lines. Hence, both methods for computing stiffness tensor $\mathbf{C}$ give equivalent results and the neural network potential generalizes from the un-strained training box to boxes under small strain. Panel **c** compares the predicted phonon density of states (PDOS) with the experiment [14] and a Stillinger-Weber potential optimized for diamond [15]. The evolution of predicted PDOS over the course of the training is shown in panel **d**.

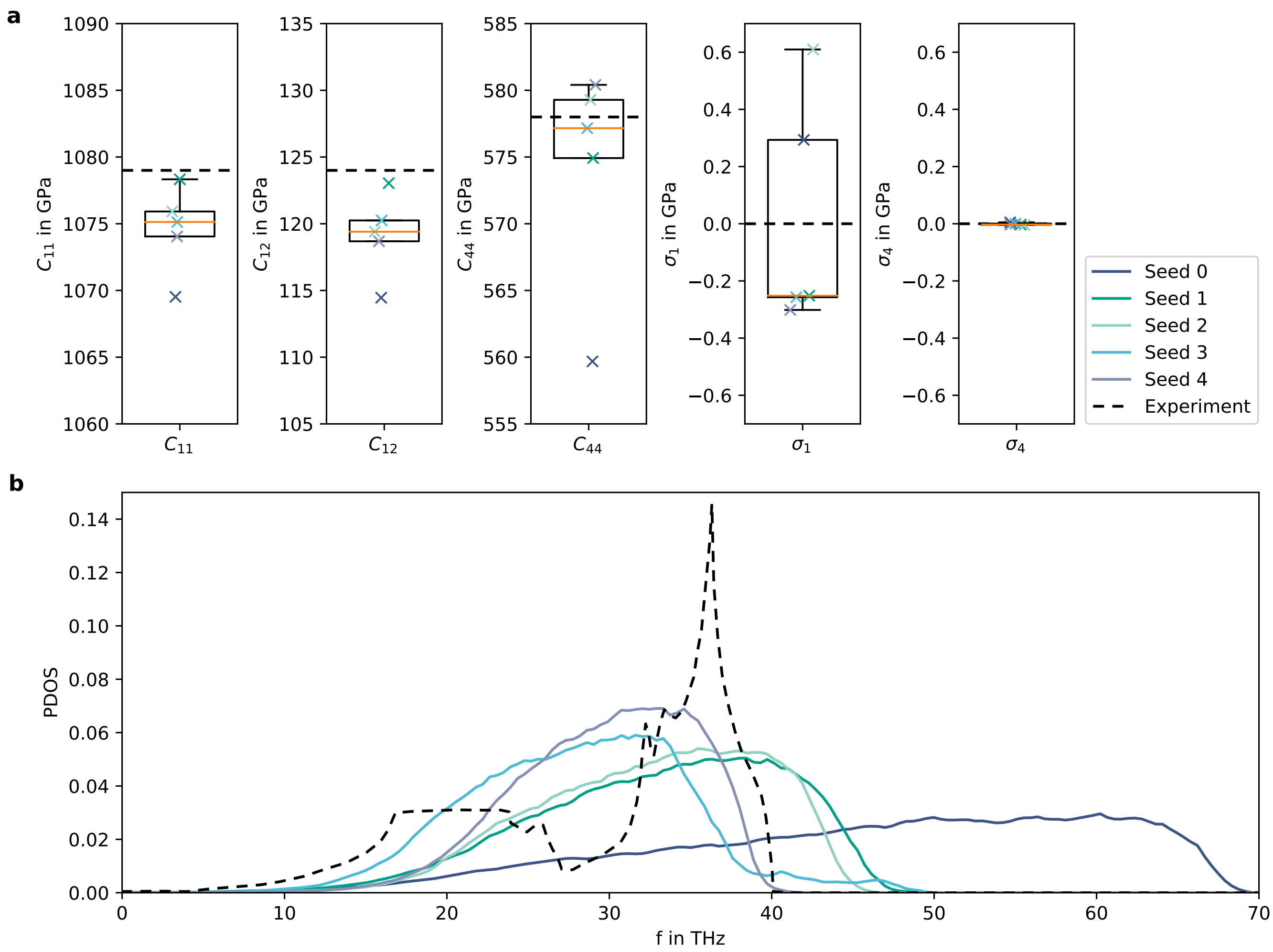

Figure 6: Random initialization study for diamond. For random seeds from 0 to 4 (controlling random initialization of neural network weights as well as initial particle velocities), the predicted observables are distributed closely around their respective targets (**a**). Corresponding predicted phonon densities of states (PDOSs) vary largely across different random seeds (**b**), confirming that different PDOSs are consistent with the target stress and stiffness values. The boxplots in **a** with median (orange line), interquartile range as box limits and whiskers representing 1.5 times interquartile range are added for visualization purposes.

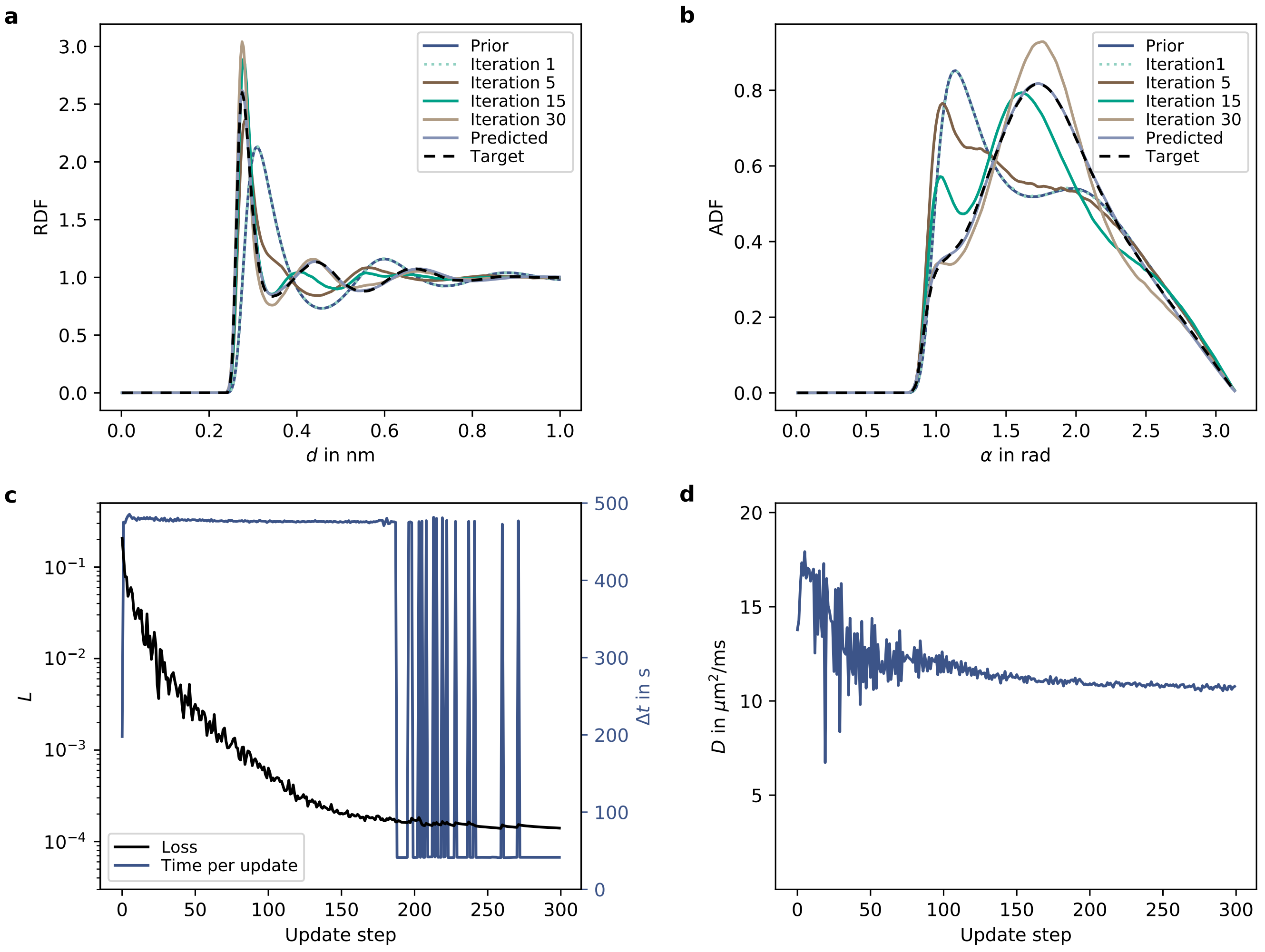

Figure 7: Supplementary results for the coarse-grained water model. Predicted radial distribution functions (RDFs) and angular distribution functions (ADFs) converge from predictions close to the prior to the respective targets (**a** - **b**). Quick reduction in the loss L confirms the learning success (**c**). Significant reduction in wall-clock time per parameter update Δt towards the end of the optimization is achieved through re-using previously generated trajectories. The predicted self-diffusion coefficient D decreases over the course of the optimization (**d**).

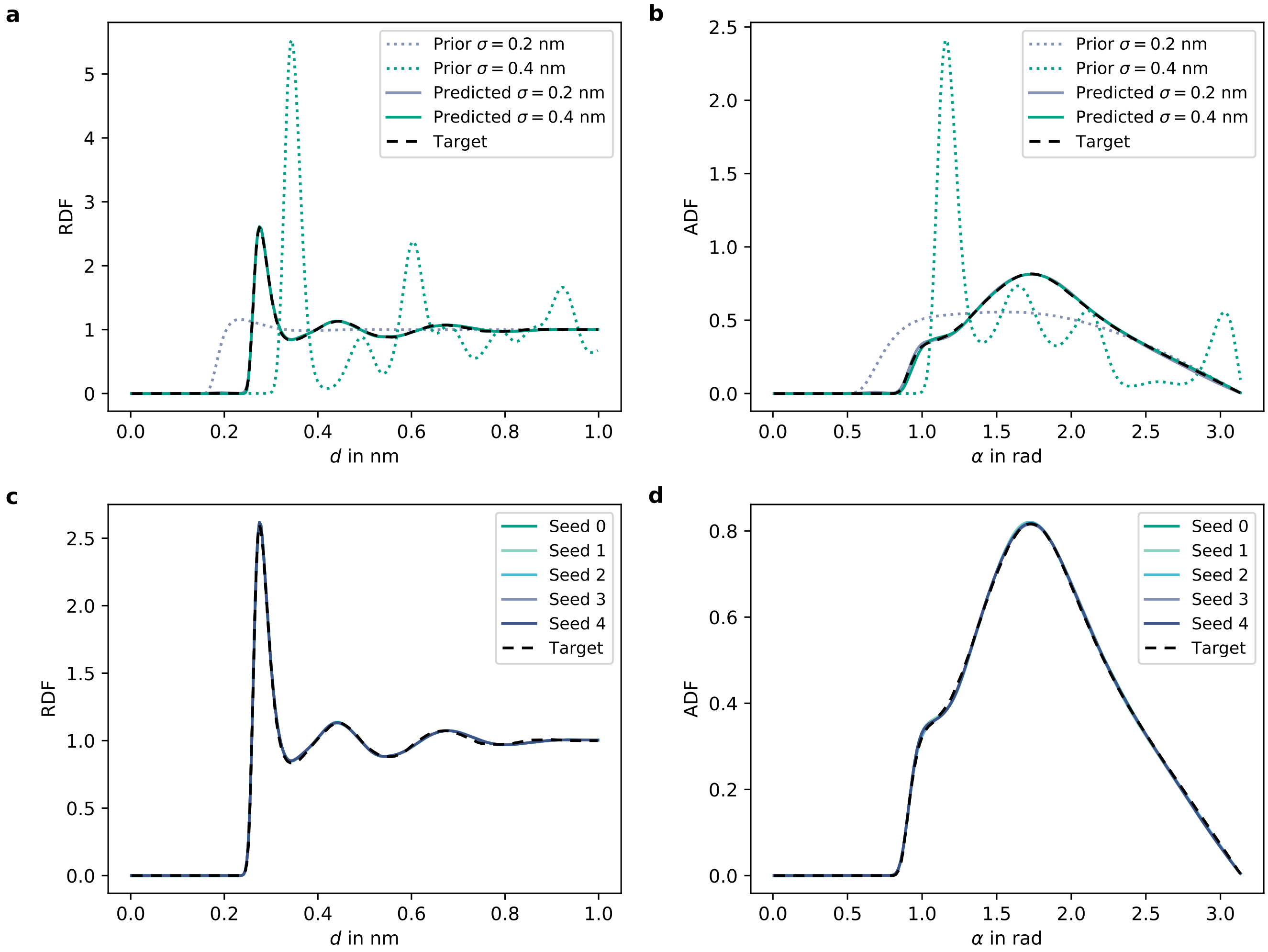

Figure 8: Robustness analysis for coarse-grained water. Predicted target observables are robust to weak choices of U^{prior} ($\mathbf{a}-\mathbf{b}$). These results are obtained using the same hyperparameters as in the reference case $\sigma_R = 0.3165$, except for longer training (1000 steps) with increased learning rate decay factor (0.25) in the case of $\sigma_R = 0.4$ nm. Additionally, predicted target observables are robust to random initialization of NN weights and initial particle velocities ($\mathbf{c}-\mathbf{d}$, $p = 68 \pm 32$ bar).

Supplementary References

[1] Kingma, D. P. & Ba, J. L. Adam: A method for stochastic optimization. In *3rd International Conference on Learning Representations, ICLR* (2015).

[2] Martyna, G. J., Klein, M. L. & Tuckerman, M. Nosé-Hoover chains: The canonical ensemble via continuous dynamics. *J. Chem. Phys.* **97**, 2635–2643 (1992).

[3] Jensen, B. D., Wise, K. E. & Odegard, G. M. The effect of time step, thermostat, and strain rate on reaxff simulations of mechanical failure in diamond, graphene, and carbon nanotube. *J. Comput. Chem.* **36**, 1587–1596 (2015).

[4] Bitzek, E., Koskinen, P., Gähler, F., Moseler, M. & Gumbsch, P. Structural relaxation made simple. *Phys. Rev. Lett.* **97**, 170201 (2006).

[5] Togo, A. & Tanaka, I. First principles phonon calculations in materials science. *Scr. Mater.* **108**, 1–5 (2015).

[6] Soper, A. K. & Benmore, C. J. Quantum differences between heavy and light water. *Phys. Rev. Lett.* **101**, 065502 (2008).

[7] Errington, J. R. & Debenedetti, P. G. Relationship between structural order and the anomalies of liquid water. *Nature* **409**, 318–321 (2001).

[8] Chen, R. T. Q., Rubanova, Y., Bettencourt, J. & Duvenaud, D. Neural Ordinary Differential Equations. In *Advances in Neural Information Processing Systems*, vol. 31 (2018).

[9] Clavier, G. *et al.* Computation of elastic constants of solids using molecular simulation: comparison of constant volume and constant pressure ensemble methods. *Mol. Simul.* **43**, 1413–1422 (2017).

[10] Klicpera, J., Groß, J. & Günnemann, S. Directional Message Passing for Molecular Graphs. In *8th International Conference on Learning Representations, ICLR* (2020).

[11] Klicpera, J., Giri, S., Margraf, J. T. & Günnemann, S. Fast and Uncertainty-Aware Directional Message Passing for Non-Equilibrium Molecules (2020). Preprint at `https://arxiv.org/abs/2011.14115`.

[12] Ramachandran, P., Zoph, B. & Le, Q. V. Searching for Activation Functions (2017). Preprint at `https://arxiv.org/abs/1710.05941`.

[13] Glorot, X. & Bengio, Y. Understanding the difficulty of training deep feedforward neural networks. In *Proceedings of the 13th International Conference on Artificial Intelligence and Statistics, AISTATS*, 249–256 (2010).

[14] Dolling, G. & Cowley, R. A. The thermodynamic and optical properties of germanium, silicon, diamond and gallium arsenide. *Proc. Phys. Soc.* **88**, 463 (1966).

[15] Barnard, A. S., Russo, S. P. & Leach, G. I. Nearest neighbour considerations in stillinger-weber type potentials for diamond. *Mol. Simul.* **28**, 761–771 (2002).
